# 2HR-Net VSLAM: Robust visual SLAM based on dual high-reliability feature matching in dynamic environments

**DOI:** 10.1371/journal.pone.0328052

**Published:** 2025-07-18

**Authors:** Wang Yang, Huang Chao, Zhang Yi, Tan Shuyi

**Affiliations:** 1 School of Computer Science and Technology, Chongqing University of Posts and Telecommunications, Chongqing, China; 2 Information Accessibility Engineering R&D Center, Chongqing University of Posts and Telecommunications, Chongqing, China; University of Marburg: Philipps-Universitat Marburg, GERMANY

## Abstract

Visual Simultaneous Localization and Mapping (VSLAM) is the key technology for autonomous navigation of mobile robots. However, feature-based VSLAM systems still face two major challenges in dynamic complex environments: insufficient feature reliability and significant dynamic interference, urgently requiring improved matching robustness. This paper innovatively proposes a dynamic adaptive VSLAM system based on the High-repeatability and High-reliability feature matching network (2HR-Net), which improves localization accuracy in dynamic environments through three key innovations: First, the 2HR feature detection network is designed, integrating the K-Means clustering algorithm into L2-Net to achieve feature point detection with both high repeatability and high reliability. Second, the lightweight YOLOv8n model is integrated to detect and remove feature points in dynamic regions in real-time, effectively reducing the impact of dynamic interference on pose estimation. Finally, the shared matching Siamese network with a unique dual-branch feature fusion strategy and similarity optimization algorithm is proposed to enhance the accuracy of feature matching. The proposed algorithm was ultimately validated using the publicly available TUM dataset. The experimental results show that the feature detection method proposed in this paper achieved a repeatability rate of approximately 70% in various dynamic scenarios, which is significantly higher than traditional methods (such as ORB-SLAM3), whose repeatability typically falls below 40%. In addition, compared with ORB-SLAM3, the root mean square error (RMSE) and standard deviation (S.D.) of the Absolute Trajectory Error (ATE) in various dynamic scenarios were reduced by approximately 90%, indicating higher localization accuracy and stability. Therefore, the experimental results demonstrate that the proposed method outperforms mainstream methods such as ORB-SLAM3 in terms of feature repeatability, matching accuracy, and localization precision, providing an effective solution for robust VSLAM in dynamic environments.

## Introduction

Simultaneous Localization and Mapping (SLAM) plays a pivotal role in the field of mobile robotics and is also a prerequisite for the realization of embodied intelligence in such robots [1]. In the past few decades, with the development of computer vision technology, Visual SLAM (VSLAM) has made great progress, and many classic methods have emerged [2]. VSLAM uses cameras as sensors to achieve autonomous localization and navigation for mobile robots. Among the existing methods, feature-based VSLAM is widely used because of its efficiency and scalability. The feature-based VSLAM localization process primarily positions mobile robots by extracting feature points and performing feature matching. The feature matching procedure discriminates the correspondence between the feature points contained in the source image and the target image, and its goal is to search for the similarities among feature point descriptors. However, the traditional feature-based VSLAM approach often suffers from localization failures in dynamic scenes due to interference caused by dynamic objects [3,4]. Therefore, the accuracies of the feature point extraction and descriptor similarity judgement steps are crucial for achieving VSLAM localization.

In recent years, many algorithms based on deep learning have been applied to visual SLAM [5,6]. For example, in the field of feature extraction, the early deep learning-based GCNv2 network was proposed. This network is compatible with ORB-SLAM2 and can easily replace its feature extraction component, thereby enhancing the performance of the system [7]. However, this network has weak generalization capabilities and can accomplish only specific tasks. Subsequently, SuperPoint was proposed, and this approach has shown significant advantages in the field of visual SLAM. This method is a deep learning-based feature point detection and descriptor generation algorithm. However, SuperPoint relies primarily on image brightness and texture information for feature extraction purposes [8]. Therefore, in dynamic environments, SuperPoint may generate erroneous feature point detection information. An R2D2 feature detection method was additionally proposed [9]. Akin to SuperPoint, R2D2 also adopts a self-supervised method to extract feature points. Unlike SuperPoint, R2D2 not only considers the repeatability of feature points but also introduces the concept of reliability. Most importantly, this method is capable of handling features at different scales, making it perform well under various viewpoints. In terms of feature matching, Sarlin *et al*. proposed the SuperGlue network [10]. The core of the SuperGlue network includes its innovative attention mechanism and graph neural network architecture, which exhibits particularly significant advantages in terms of handling relative pose estimation tasks in extreme cases. Although SuperGlue is a powerful feature matching tool, it has several limitations in terms of its computational cost and memory consumption. Similarly, the AMatFormer model and the SL-SLAM method also employ deep learning for feature matching. The AMatFormer model first enhances the information of its descriptor through self-cross attention and then performs feature matching through a shared FFN module [11]. SL-SLAM combines deep learning algorithms such as SuperPoint and LightGlue to improve the matching accuracy and algorithmic robustness of SLAM systems in various environments [12–14]. However, they all have some limitations, including the need for computational resources and generalizability in new environments. Moreover, some algorithms may fail to accurately match feature points in complex scenes.

Inspired by the above methods, we propose a 2HR-Net VSLAM system. First, to ensure the quality of the feature point extraction process, we screen the feature points with high reliability and high repeatability. Second, the feature points located within the dynamic object box are removed. Then, we propose shared matching Siamese networks to find matching feature pairs by judging the similarity of their descriptors. The specific main contributions are summarized as follows.

- Firstly, to select feature points with high reliability and repeatability, we drew inspiration from the R2D2 feature extraction method and chose L2-Net as the backbone network for feature point extraction. This network extracts feature points that are both reliable and repeatable. Higher reliability enhances the accuracy of feature point matching, while higher repeatability increases the likelihood of detecting these points across different images. To identify such feature points, we integrated the K-means clustering method into the L2-Net backbone network. We denote these feature points as 2HR (High-Reliability and High-Repeatability Feature Points).
- Secondly, to eliminate the interference of feature points on dynamic objects, we used YOLOv8n to detect and label dynamic objects, and subsequently removed the feature points within the regions occupied by these objects.
- Thirdly, in order to improve the feature matching rate, we propose a Siamese neural network. This network embeds the descriptors of the 2HR after removing dynamic interference points in two images into the same network layer for processing. Subsequently, the similarity between these descriptors is evaluated using a sigmoid function. Finally, feature matching pairs are searched for based on the similarity value.
- Finally, the 2HR-Net VSLAM system is proposed. To validate its robustness and accuracy, we conducted a series of experiments on the TUM dataset.

The remainder of this paper is organized as follows. In the related work section, we review the existing literature on feature point detection and feature matching algorithms. The methods section provides a detailed explanation of the specific method design. First, the overall design of the 2HR-Net network and the dynamic object detection method of YOLOv8n are presented. The improved feature extraction method and the shared Siamese network are subsequently highlighted. Finally, the framework of 2HR-Net VSLAM is given and explained. In the experiments section, we conduct experiments on multiple sequences derived from the TUM dataset and present the experimental results. The final section summarizes the paper and proposes idea for our future work.

## Related work

### Feature point extraction

Traditional methods such as SIFT [15], SURF [16], FAST [17], and ORB [18] have long been used to detect initial features. However, these methods often struggle in complex dynamic scenes. In response to these challenges, researchers have pursued two main avenues of improvement. On one hand, efforts have been made to enhance positioning accuracy through the fusion of multiple sensors. On the other hand, the advent of neural networks has significantly boosted the ability to detect feature points [19].

Early advancements in this field include the proposal of the L2-Net structure, which aimed to enhance the original feature point extraction model [20]. L2-Net is a deep learning-based method that leverages a Convolutional Neural Network (CNN) to learn descriptors of local image patches in Euclidean space. These descriptors are widely used for computer vision tasks such as image matching, 3D reconstruction, and camera positioning. Building on this foundation, GeoDesc was introduced as a method to learn local descriptors by integrating geometric constraints into the multiview reconstruction process [21]. By incorporating geometric constraints in data generation, sampling, and loss calculation, GeoDesc has demonstrated strong performance in various large-scale benchmark tests. In the same year, the well-known SuperPoint approach was proposed. SuperPoint is a self-supervised framework capable of both local feature detection and description. Through self-supervised training, it simultaneously generates feature point locations and descriptors. R2D2 also employs a self-supervised learning network, emphasizing the repeatability and reliability of feature points. It can stably detect feature points and generate reliable local descriptors under varying viewing angles and lighting conditions. HF-Net, proposed in 2019, is a hierarchical localization method based on a holistic CNN. It can simultaneously predict both local features and global descriptors [22]. However, its performance is highly dependent on the given dataset. If the training data do not adequately represent real-world scenarios, the model’s generalization ability may be compromised. Similarly, the ASL-Feat method can generate descriptors while detecting feature points [23]. It accepts images of any size as input. However, the generated descriptors are 128-dimensional floating vectors, which are not ideal for feature matching. To address this limitation, BASL-AD was proposed in 2024 [24]. This method employs a descriptor designed based on binary deep learning. However, it takes a longer time to extract BASL, which may limit its practicality in real-time applications.

### Feature matching algorithm

The goal of feature matching is to identify the corresponding points between two frames. Initially, the feature matching process in Visual Simultaneous Localization and Mapping (VSLAM) relied on brute-force matching, which was not only time-consuming but also prone to significant errors. To address these limitations, researchers introduced the nearest-neighbour matching method, which effectively reduced the computational time required for matching.

With the advent of deep learning, researchers demonstrated that integrating deep learning-based matching methods into VSLAM systems could significantly enhance both matching speed and accuracy. For instance, D2-Net is a trainable convolutional neural network that serves as both a dense feature descriptor and a feature detector. However, its feature detection accuracy is relatively low [25]. In 2020, SuperGlue was introduced, leveraging graph neural networks and optimal transport theory to achieve robust feature point matching. The introduction of SuperGlue marked a major milestone in the evolution of VSLAM algorithms towards end-to-end deep learning. Inspired by SuperGlue, OpenGlue was proposed in 2022 as an open-source feature matching framework based on graph neural networks. However, OpenGlue may be limited to specific datasets [26].

In 2021, LoFTR was proposed as a local feature matching method based on the Transformer architecture [27]. LoFTR can directly output matched pairs of points between images without the need for a detector. This method first extracts coarse and fine features using a Convolutional Neural Network (CNN) and a Feature Pyramid Network (FPN), respectively. The features are then refined through positional encoding and the self-attention and cross-attention layers in the Transformer, completing the matching process in a coarse-to-fine manner. However, this method faces efficiency limitations due to the execution of the Transformer over the entire coarse feature graph, resulting in large token sizes.

## Methods

### The overview of 2HR-Net

**Fig 1:**
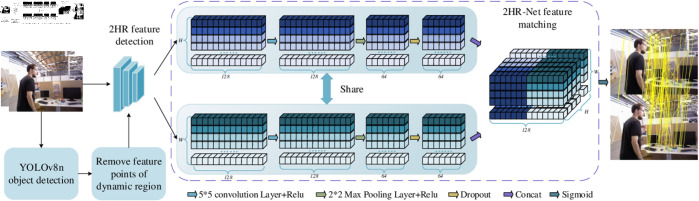
The framework of 2HR-Net.

The framework of the 2HR-Net is illustrated in Fig 1. The core challenges of the 2HR-Net network structure revolve around two key aspects: (1) how to effectively extract highly robust feature points and (2) how to optimize the feature matching algorithm to achieve improved matching accuracy. Under the premise that all individuals appearing in the images have been informed and have consented, this study, inspired by the L2-net feature extraction network in R2D2, proposes a feature extraction method with high reliability and repeatability, named 2HR feature detection. The specific details of feature detection will be elaborated in detail in the “2HR feature detection” section.

In terms of feature matching, this paper employs a shared Siamese network for feature matching, called 2HR-Net feature matching, as shown in the purple dotted box in Fig 1. This approach aims to enhance the accuracy of the feature matching process through an advanced algorithm design. Specifically, this module first performs convolution on the feature descriptor with a length of 128 to enhance the expressiveness of the features. Subsequently, the length of the feature descriptor is compressed to 64 through the max pooling operation of feature, thereby reducing the computational complexity while retaining key information. In order to improve the generalization performance of the model and avoid overfitting, we further introduced a Dropout layer. The feature length after processing by this layer remains unchanged at 64. Next, we borrowed the idea of the twin network and used the concat function to merge the feature representations of the two images. The length of the merged feature increased to 128. At this point, the feature matching problem was transformed into a classification task. The core of feature matching is to evaluate the similarity of feature points in two frames of images, so it is essentially a binary classification problem. Therefore, we chose the Sigmoid function to evaluate the similarity between feature points. The output value of the Sigmoid function is between 0 and 1. The closer the value is to 0, the more dissimilar the feature points are, while the closer the value is to 1, the more similar the feature points are. Based on this criterion, we finally only retain those feature point matching pairs whose output result of the Sigmoid function is 1. The structure of the shared Siamese network will also be described in detail in the “2HR-Net feature matching” section.

**Fig 2:**
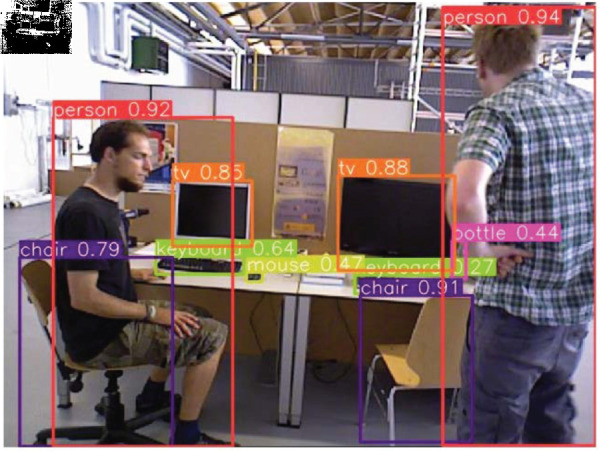
The result of the object detection using YOLOv8n.

On the other hand, in order to avoid the interference caused by dynamic objects, this study first applies the YOLOv8n [28] algorithm for object detection, and the detection result is detailed in Fig 2. The detection results show that YOLOv8n can not only successfully detection larger objects (e.g., people, chairs, etc.), but also successfully detect relatively smaller objects (e.g., keyboards, mice, books, etc.). Therefore, the use of the YOLOv8n method for semantic segmentation in VSLAM is consistent with the needs of VSLAM applications. Subsequently, the results of object detection will be classifying the dynamic characteristics. In order to identify the dynamic characteristics of different categories of objects, this paper carries out a preliminary classification based on observations in daily life, and the classification results are shown in Table 1. In addition, because this paper mainly employs the TUM dataset for experimental validation, we only exclude the feature points on the ‘high-dynamic objects’ in the subsequent study.

**Table 1:** Classification of the dynamic properties of common objects in life.

Classification	Objects
Highly Dynamic	People
Medium Dynamic	Chairs, Books
Low Dynamic	Desks, TVs

### 2HR feature extraction

**Fig 3:**
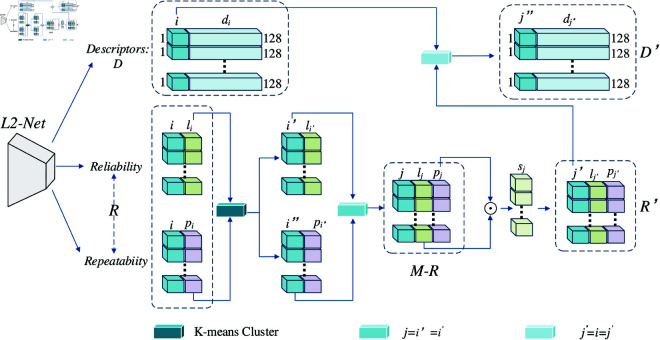
The flowchart of 2HR feature extraction.

In the field of VSLAM, the core task of feature matching is to detect and match feature points between two consecutive keyframes. Therefore, it is very important to detect and extract the highly quality feature points in the two frames of images for the accuracy of feature matching. In this context, a higher repeatability score indicates a greater probability of feature points appearing in the subsequent frame, while a higher reliability score signifies superior quality of the feature points. Since R2D2 method can filter feature points by calculating reliability scores and repeatability scores, we use the feature extraction method of R2D2 as the main network to extract feature points. In this context, the backbone network for feature point extraction in R2D2 is the L2-Net backbone, which outputs a descriptor tensor and two score tensors respectively. In order to find the feature points with high reliability and high repeatability, we have improved the L2-Net backbone. The improved framework is shown in Fig 3.

First, we define the set of feature points obtained by L2-Net backbone as follows:

F={D,R} (1)

$$𝐷=\left\{\left(i,d_{i}\right),i\in N,N=\alpha \right\}$$ (2)

$$𝑅=\left\{\left(i,l_{i},p_{i}\right),i\in N,N=\alpha \right\}$$ (3)

where, *F* represents the set of feature points, *D* represents the descriptors, and *R* represents the set of reliability and repeatability scores. *i* represents the index number of feature points, α represents the number of extracted feature points, *l*_*i*_ represents the reliability score of the ${\text{i}}^{\text{th}}$ point, *p*_*i*_ represents the repeatability score of the ${\text{i}}^{\text{th}}$ point.

In the process of feature point extraction, directly selecting feature points with high reliability and high repeatability from a large number of feature points presents numerous challenges. On one hand, individually assessing the reliability and repeatability of each feature point is highly inefficient. On the other hand, the distribution patterns of feature points are complex, and simple threshold-based screening methods are insufficient for effective differentiation. Clustering, as an unsupervised machine learning algorithm, can partition data into several clusters such that the similarity within each cluster is high, while the similarity between clusters is low. Therefore, in feature point extraction, clustering methods can be employed to group feature points based on the similarity of their reliability and repeatability scores, thereby more effectively identifying groups of feature points with high reliability and repeatability. This provides a more rational basis for subsequent feature point selection.

Among various clustering algorithms, the K-means algorithm is distinguished by its low time complexity. It can complete the clustering task for large-scale feature point data in a relatively short period of time, thereby meeting the demands of visual simultaneous localization and mapping (VSLAM) systems that require high real-time performance in feature point extraction. Additionally, this method allows for the flexible selection of the number of clusters k based on specific needs, which helps achieve the optimal clustering effect. The clustering results are also easy to interpret and understand, facilitating subsequent analysis of the clustering outcomes. Therefore, in this study, the K-means method is employed to cluster the reliability and repeatability scores *l*_*i*_ and *p*_*i*_ of feature points separately. In the experiments, we chose *k* = 3, that is, each set of feature points was divided into three categories, and the group with the highest scores was selected as the final feature point data.

Secondly, to further improve the quality of feature points and provide more reliable inputs for subsequent feature matching and VSLAM systems, we obtained the clustering result from *M* – *l* and *M* – *p*. The definitions for *M* – *l* and *M* – *p* are as follows:

$$M-l=\left\{\left(i^{\prime },l_{i^{\prime }}\right),i^{\prime }\in N,N<\alpha \right\}$$ (4)

$$M-p=\left\{\left(i^{\prime \prime },p_{i^{\prime \prime }}\right),i^{\prime \prime }\in N,N<\alpha \right\}$$ (5)

Then, to obtain feature points with high reliability and high repeatability, we identify the set of feature points *M* – *R* that have the same index in both *M* – *l* and *M* – *p*. The definition is as follows:

$$M-R=\left\{\left(j,l_{j},p_{j}\right),j=i^{\prime }=i^{\prime \prime },j\in N<\alpha \right\}$$ (6)

To further eliminate feature points that, despite having high reliability or repeatability, still have an overall unsatisfactory quality, we multiply the reliability score by the repeatability score to obtain a composite score *s*_*j*_, and the set of score values is expressed as *S* – *R*. Subsequently, feature points with lower score values are filtered out using a threshold *T*, resulting in the refined set $R^{\prime }$. *S* – *R* and $R^{\prime }$ are defined as follows:

$$S-R=\left\{\left(j,s_{j}\right),s_{j}=l_{j}*p_{j},j\in N<\alpha \right\}$$ (7)

where *s*_*j*_ represents the score value of the ${\text{j}}^{\text{th}}$ feature point.

$$R^{\prime }=\left\{\left(j^{\prime },l_{j^{\prime }},p_{j^{\prime }}\right),j^{\prime }<j\right\}$$ (8)

Finally, we identify the descriptors $D^{\prime }$ of feature points with high reliability and repeatability by matching indices. And the final set of feature points with high reliability and repeatability is obtained as $F^{\prime }$. The $D^{\prime }$ and $F^{\prime }$ are defined as follows:

$$D^{\prime }=\left\{\left(j^{\prime \prime },d_{j^{\prime \prime }}\right),j^{\prime \prime }=j^{\prime }=i\in N<\alpha \right\}$$ (9)

$$F^{\prime }=\left\{D^{\prime },R^{\prime }\right\}$$ (10)

### 2HR-Net feature matching

For every feature point in the image, there is a descriptor to describe its local features. These local features are used to represent the attributes of the feature points, so as to complete the task of feature matching between different feature points. In this paper, the descriptor is a three-dimensional tensor of H×128, where *H* represents the number of feature points in the image. In other words, each descriptor is a 128-dimensional vector. Therefore, we define the descriptors as follows:

$$D^{\prime }=[\begin{array}{c} \left[\begin{array}{cccc} d_{0,0} & d_{0,1} & \ldots & d_{0,w-1} \end{array}\right]\\ \left[\begin{array}{cccc} d_{1,0} & d_{1,1} & \ldots & d_{1,w-1} \end{array}\right]\\ \ldots \\ \ldots \\ \left[\begin{array}{cccc} d_{h-1,0} & d_{h-1,1} & \ldots & d_{h-1,w-1} \end{array}\right] \end{array}]$$ (11)

$$d=[\begin{array}{cccc} x_{0} & x_{1} & \ldots & x_{127} \end{array}]$$ (12)

where $D^{\prime }$ denotes the descriptor structure of the entire image, while *d* signifies the descriptor of an individual feature point.

The feature point matching usually uses distance measurement to compare the similarity between two groups of feature vectors. Specifically, the distance between feature points is the distance between descriptors. In general, the matching method usually use Nearest Neighbor Search, K-Nearest Neighbor and or other more advanced methods to find the nearest descriptor, so as to determine the corresponding relationship between feature points.

**Fig 4:**
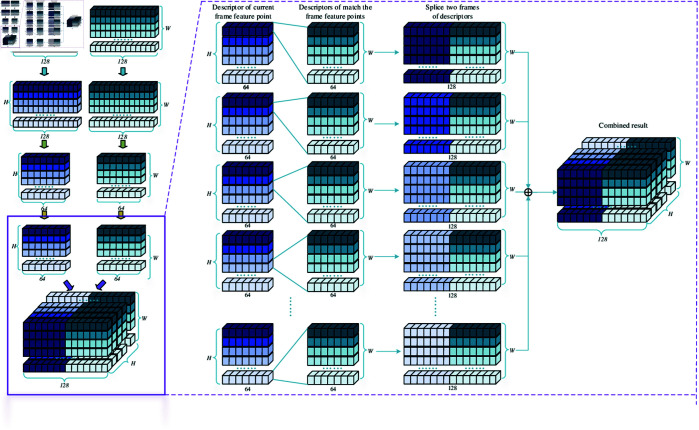
The detailed process of the “Concat” program.

To enhance the feature matching rate and reduce the number of model parameters, this paper proposes a feature matching algorithm based on shared Siamese network architecture, which we refer to as the 2HR-Net network. The algorithm processes descriptors through two identical sub-networks that share weights and evaluates their similarity by comparing the processed descriptors. The overall structure of the 2HR-Net network is shown within the purple dashed box in Fig 1. For a more detailed explanation, the detailed process of the “Concat” procedure is illustrated in Fig 4. After undergoing 2HR structural screening, the descriptors obtained are processed through a series of operations, resulting in a structure of N×64. Here, *N* represents the number of feature points, with each row corresponding to the descriptor of one feature point. During the concatenation process, the descriptor of each feature point in the current keyframe is concatenated with the descriptor of the matching keyframe, ultimately forming a three-dimensional structure of H×W×128. Finally, the Sigmoid function is applied to the three-dimensional structure to evaluate the similarity between feature points.

To provide a clearer explanation of the processing flow of the 2HR-Net feature matching proposed in this paper, we will use the descriptor of a feature point as an example for illustration and explanation.

First, we perform convolution operations on the descriptor *d*. To extract the most salient features from the descriptor, we employ max pooling. This process transforms *d* into $d^{\prime }$, as represented below:

$$d^{\prime }=[\begin{array}{cccc} x_{0}^{\prime } & x_{1}^{\prime } & \ldots & x_{63}^{\prime } \end{array}]$$ (13)

Secondly, to prevent overfitting and enhance the model’s generalization capability, we introduce Dropout layers [29] into our network. Then, because the core of feature matching lies in evaluating the similarity of two feature points, therefore, based on the principle of shared Siamese network [30,31], the similarity problem is transformed into a classification problem. Subsequently, the descriptors $d^{\prime }$ of two feature points are fused through the concat method [32,33] to obtain $d^{\prime \prime }$, which is expressed as follows:

$$d^{\prime \prime }=[\begin{array}{cccc} x_{0}^{\prime \prime } & x_{1}^{\prime \prime } & \ldots & x_{127}^{\prime \prime } \end{array}]$$ (14)

Finally, when addressing the classification task, we treat it as a binary classification problem. In order to better fit positive and negative samples and independence evaluation, we introduced Sigmoid [34] activation function to obtain the optimal positive sample value in the prediction process. The Sigmoid calculation formula is defined as follows:

$$\sigma \left(\chi \right)=\frac{1}{1+e^{-\chi }}$$ (15)

where σ represents the Sigmoid function and χ is the input value, with $\chi =d^{\prime \prime }$.

### 2HR-Net VSLAM

**Fig 5:**
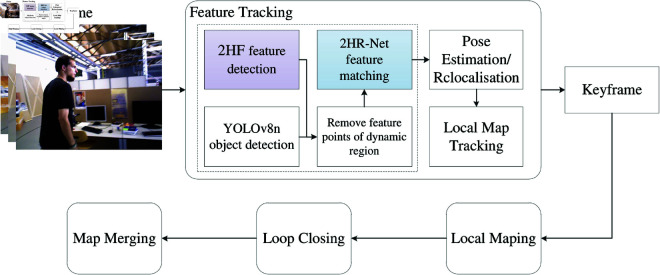
The framework of the 2HR-Net VSLAM network.

The detailed framework of the 2HR-Net VSLAM network is shown in Fig 5. First, we construct the 2HR-Net network. In this network, the 2HR model is applied to extract feature points with high reliability and high repeatability. Next, the feature points in the dynamic object region are excluded by integrating the object detection results derived from YOLOv8n. Then, the descriptors of the filtered feature points are input into the shared Siamese network, and their similarity is calculated. Following this, we apply the 2HR-Net network to the front-end visual odometry of a VSLAM system. Through this integration scheme, 2HR-Net network can effectively provide accurate visual information for the positioning and navigation of mobile robots, thus enhancing the performance and reliability of VSLAM systems.

## Experiment

In this section, we train and test the model on a dataset provided by the Technical University of Munich (Technische Universität München, TUM) [35]. In addition, we evaluate the performance of the feature point extraction process, the feature matching strategy, and the 2HR-Net VSLAM system through a series of qualitative experiments.

### Model datasets

We utilized the “freiburg3_sitting_xyz” subset from the TUM dataset for our model training and validation. This subset comprises 1261 images, which we refer to as “fre3/s/xyz.” Specifically, 80% of the fre3/s/xyz data was allocated to the training set, while the remaining 20% was used as the validation set.

To extract high-quality feature points, we first employed an improved feature extraction network to detect feature points in the images. Subsequently, we manually annotated the matching results in the fre3/s/xyz dataset. More precisely, for each pair of feature points, if they were a match in the real-world scenario, they were labeled as positive samples; otherwise, they were labeled as negative samples. Through this process, we collected a total of 42,565 pairs of matching data, comprising both positive and negative samples.

### Evaluation datasets

The TUM dataset is a widely used RGB-D (colour image and depth image) dataset published by the Computer Vision Lab at Technische Universität München (TUM) in Germany. The dataset contains a wide range of scenarios, such as offices and home environments. It covers different levels of dynamics, from static scenes to highly dynamic scenes. Therefore, the TUM dataset is widely used to test and evaluate various SLAM algorithms. In particular, its highly dynamic subdataset contains several specifically designed scenarios that are often used to evaluate the performance of SLAM algorithms in dynamic environments. Therefore, in this experiment, we use the highly dynamic subdataset of the TUM dataset to conduct an evaluation. The high-dynamic dataset consists of four sequences, namely “freiburg3_walking_halfsphere” (referred to as fre3/w/half), “freiburg3_walking_xyz” (referred to as fre3/w/xyz), “freiburg3_walking_rpy” (referred to as fre3/w/rpy), and “freiburg3_walking_static” (referred to as fre3/w/sta).

### Evaluation of 2HR feature extraction

In this section, we systematically validated the effectiveness and superiority of the improved feature point selection method through K-means clustering parameter experiments and ablation studies on feature extraction.

#### K-means clustering parameter study.

The feature point selection method based on K-means clustering results not only considers the quality of individual feature points but also takes into account the distribution characteristics of the group of feature points, thereby being able to more accurately identify high-quality feature points. Compared with the traditional method that relies solely on threshold values for selection, the clustering-based selection method can better adapt to the distribution characteristics of feature point data, significantly improving the accuracy and robustness of feature point selection.

**Fig 6:**
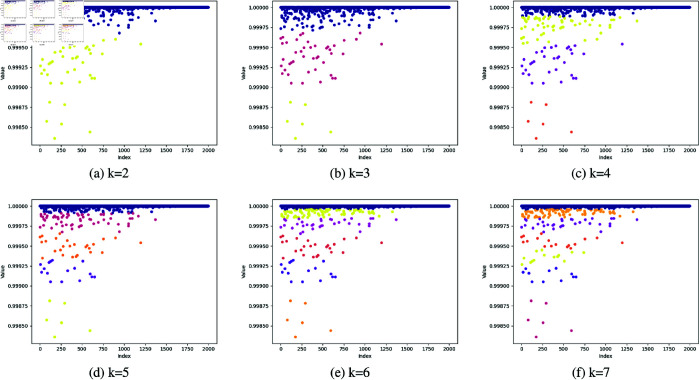
Experimental results of different parameters k in K-Means algorithm for reliability.

**Fig 7:**
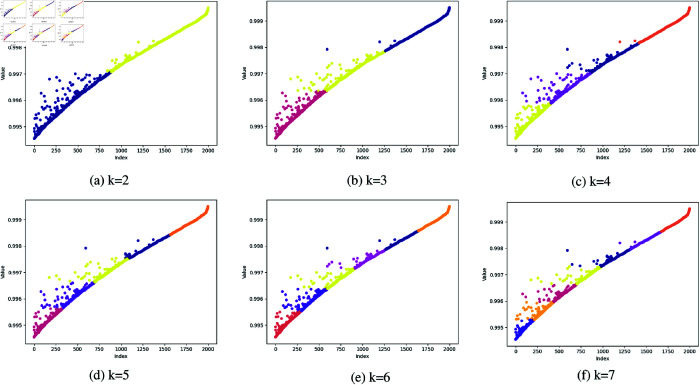
Experimental results of different parameters k in K-Means algorithm for repeatability.

In the experiments, we conducted a systematic investigation and optimization of the number of clusters k in the K-means clustering algorithm. Specifically, In the reliability clustering experiment, we performed clustering analyses for k values ranging from 2 to 10, with the clustering results for k values from 2 to 7 illustrated in the Fig 6. By comparing the clustering outcomes across different k values, it was observed that when *k* = 2, the clustering results were relatively dispersed; whereas for *k* > 3, the clustering performance deteriorated. In contrast, the optimal clustering performance was achieved when *k* = 3, with the most rational distribution of feature points. Therefore, in this experiment, we selected *k* = 3. Similarly, in the clustering experiments with high reproducibility, the clustering results for different values of k are shown in Fig 7. As can be seen from the figure, the clustering result is relatively better when *k* = 2. Therefore, in clustering experiments with high reproducibility, we select *k* = 2 as the optimal clustering parameter. Under this condition, the selected feature points demonstrated higher reliability and repeatability in subsequent applications.

#### Ablation study of 2HR feature extraction.

In order to evaluate the quality of feature points, we conducted comparative experiments on highly datasets of TUM, and evaluated the repetition rate (%Rep.) and accuracy rate (%Prec.) of feature points. The statistical results are shown in Table 2. Repetition rate and accuracy rate are important evaluation metrics in feature extraction tasks, which are used to evaluate the accuracy and reliability of feature extraction algorithms. The repetition rate measures the proportion of feature pairs successfully matched in the shared view to the total detected feature points, which directly reflects the ability of the algorithm to recognize the same feature points. The accuracy rate refers to the ratio of the logarithm of correctly matched feature points to the total number of matched feature points. The higher the precision, the greater the proportion of correct matches in the matching results, and the smaller the proportion of incorrect matches.

**Table 2:** Performance comparison of different feature extraction methods.

Dataset		FAST	SURF	ORB	SuperPoint	D2-Net	R2D2-Net	LoFTR	Aslfeat	Ours
Decriptors Type		128-F	64-F	256-B	256-F	512-F	128-F	256-F	128-F	128-F
f3/w/rpy	%Rep.	36.43%	21.69%	41.78%	40.73%	46.52%	64.45%	67.36%	57.73%	71.62%
	%Prec.	98.21%	98.62%	99.18%	99.13%	98.17%	98.94%	99.15%	99.02%	99.38%
f3/w/xyz	%Rep.	48.66%	47.02%	24.84%	49.94%	51.77%	67.48%	67.94%	41.84%	72.07%
	%Prec.	98.37%	98.98%	99.34%	99.04%	98.79%	98.19%	99.40%	99.26%	99.63%
f3/w/sta	%Rep.	63.33%	59.34%	33.87%	55.29%	57.71%	71.28%	72.69%	69.07%	74.61%
	%Prec.	98.41%	99.16%	99.12%	99.43%	98.01%	98.97%	99.01%	98.55%	99.42%
f3/w/half	%Rep.	46.33%	45.01%	23.33%	39.28%	40.04%	56.77%	56.91%	54.83%	69.69%
	%Prec.	98.26%	98.79%	99.31%	99.04%	98.33%	98.81%	99.11%	98.91%	99.43%

From the analysis of Table 2, in terms of the traditional method, the ORB feature point detection method has poor recall but good precision on the dataset. This may be related to the structure of its feature point descriptors. The descriptors of ORB feature points are a 256-dimensional binary array, while the descriptors of other methods are all floating-point data. Therefore, the probability of ORB correctly matching is relatively high during feature matching. However, the repetition rate is low and the accuracy is high, which means that a large number of feature points detected by ORB will be wasted during the feature matching process. Overall, the proposed method has a significantly higher repetition rate and a higher accuracy than the traditional methods. On the other hand, the R2D2 framework has advantages in improving the repetition rate of feature points. Therefore, compared with deep learning methods, the method proposed in this paper not only ensures high accuracy, but also shows general superiority in repetition rate. At the same time, in the process of feature matching, the dimension of descriptor not only affects the accuracy rate but also affects the storage space of feature points. The descriptors proposed in this paper are 128-dimensional floating-point data, so they do not occupy a large amount of storage space during the feature matching process. In summary, the method proposed in this paper has good competitiveness, whether compared with traditional methods or deep learning methods.

### Ablation study of 2HR-Net feature matching

In the feature matching experiment evaluation, we compared the model proposed in this paper with other deep learning models, including SuperGlue, D2-Net, and LoFTR. And the average matching rate (%M.S.) and average matching accuracy (%MMA) were calculated on four highly dynamic datasets of TUM to evaluate the experimental effect. Average matching rate (%M.S.) and average matching accuracy (%MMA) are important evaluation indexes in feature matching tasks, which are used to evaluate the reliability and accuracy of feature matching algorithms. The average matching rate (%M.S.) is a measure of the ratio of the logarithm of correctly matched feature points to the total logarithm of feature points, which is usually used to quantify the accuracy of feature matching.

During the statistical process, for each matched point pair, we first find its corresponding point on the other image through a single correspondence transformation, and then calculate the distance between them. Then we set a threshold, and if the distance between the matching points is less than this threshold, it will be counts as a correct match. The mean match accuracy (%MMA) is a measure of the proportion of correct matches in a match pair based on the match result. In the statistical analysis process, we first obtain the results of the feature matching, and then use the geometric consistency method to identify the correct matching pair. The final statistical results are shown in Table 3. From the analysis in Table 3, it can be found that in terms of average matching rate (%M.S.), the method proposed in this paper has better improvement than other methods. In terms of the mean matching accuracy (%MMA), the proposed method and LoFTR method are overall better than the other two methods.

**Table 3:** Performance comparison of different VSLAM methods: matching rate (%M.S.), mean matching accuracy (%MMA).

Datasets		SuperGlue	D2-Net	LoFTR	Ours
f3/w/rpy	%M.S.	20.83%	18.32%	23.42%	24.88%
	%MMA	52.38%	51.37%	54.23%	54.79%
f3/w/xyz	%M.S.	23.27%	19.59%	24.30%	27.30%
	%MMA	57.46%	56.83%	59.23%	59.48%
f3/w/sta	%M.S.	21.47%	17.66%	22.47%	24.57%
	%MMA	62.94%	63.95%	63.66%	63.86%
f3/w/half	%M.S.	25.24%	23.47%	27.46%	30.23%
	%MMA	55.65%	54.14%	57.70%	88.30%

**Fig 8:**
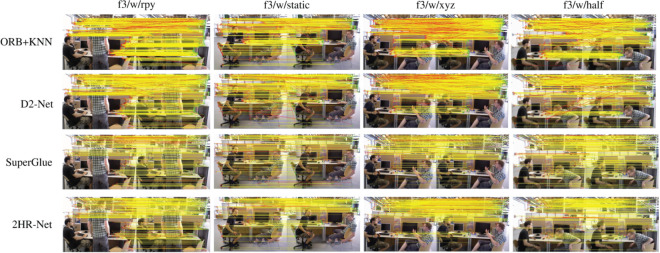
Feature matching results of different methods.

In this section, we use the highly dynamic dataset of TUM to verify the image matching performance of local features. We visualized the feature matching results of ORB combined with Nearest Neighbour Matching (KNN), D2-Net, SuperGlue and the method proposed in this paper. D2-Net is a convolutional neural network model for joint detection and description of local image features. D2-Net is able to perform the feature extraction task end-to-end with better performance and efficiency than traditional step-by-step methods. The SuperGlue utilizes self-attention and cross-attention mechanisms to match feature points based on their location and their visual appearance, so that SuperGlue can achieve significant performance improvements in feature matching. The visualization results are shown in Fig 8, where the yellow lines represent correct matches and the red lines indicate incorrect matches. It can be seen from the Fig 8 that the method proposed in this paper can accurately match most of the feature points. Compared with other methods, it intuitively proves that our proposed shared Siamese network is more effective in the feature matching task.

### Evaluation of 2HR-Net VSLAM system

In order to evaluate the effectiveness of the system proposed in this paper, this section presented a quantitative evaluation of ORB-SLAM3 and 2HR-Net VSLAM in terms of Absolute trajectory error (ATE) [36,37]. In addition, we also compared and analyzed their trajectory results. The ATE is used to evaluate the absolute accuracy of trajectory localization, which is the difference between the robot’s trajectory during operation and the real trajectory. Where, the Root Mean Square Error (RMSE) and Standard Deviation (S.D.) of the ATE are often used to evaluate the accuracy and stability of the SLAM system.

**Fig 9:**
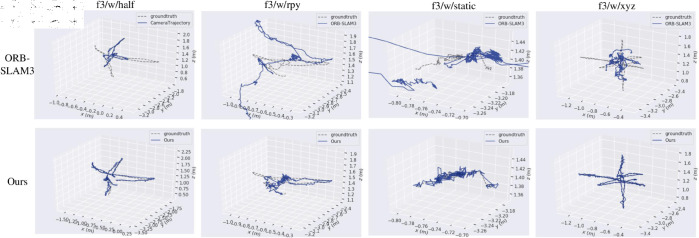
The trajectory comparison results of different SLAM.

The evaluation results are shown in Table 4. The Table 4 shows a comparison of the ATE performance, including RMSE and S.D., between ORB-SLAM3 and 2HR-Net VSLAM on the four datasets. At the same time, the table also lists the relative improvements of the proposed system compared to ORB-SLAM3 on these metrics. It can be seen from the data in Table 4 that the performance of the 2HR-Net VSLAM has a greatly improved compared with ORB-SLAM3 under four sets of dynamic sequences. Therefore, it is proved that the proposed system can significantly improve the positioning performance of mobile robots in dynamic environments. At the same time, we also did trajectory comparison experiments on four datasets. The experimental results are shown in Fig 9, where the dashed line represents the real trajectory of the dataset, and the blue solid line represents the running trajectory. From the trajectory comparison graph in Fig 9, it can be seen that the trajectory of 2HR-Net VSLAM is more closely matched to the real trajectory than that of ORB-SLAM3. Therefore, it is proved that the SLAM system proposed in this paper has better robustness in dynamic environments.

**Table 4:** The ATE evaluation values of different SLAM.

Datasets	ORB-SLAM3		2HR-Net SLAM		Improvements(%)	
	RMSE	S.D.	RMSE	S.D.	RMSE	S.D.
f3/w/rpy	3.352	0.556	0.214	0.037	93.61	93.34
f3/w/xyz	3.587	0.284	0.121	0.036	96.62	87.32
f3/w/sta	3.483	0.551	0.173	0.019	95.03	96.55
f3/w/half	3.456	0.238	0.288	0.023	91.66	90.33

The method proposed in this paper introduces optimization techniques in the feature detection and feature matching stages. Although these improvements lead to a slight increase in runtime compared to the traditional ORB-SLAM3 method (approximately 150 ms), the overall runtime remains within an acceptable range and does not affect the system’s performance in practical applications. Moreover, the proposed method employs a lightweight shallow neural network model that does not rely on additional hardware support. Therefore, the VSLAM system presented in this paper demonstrates excellent performance in terms of real-time capability and resource requirements, effectively meeting the needs for real-time localization and mapping in dynamic environments.

### Real scenario evaluation

**Fig 10:**
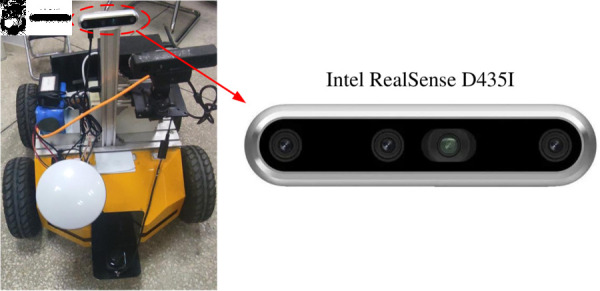
The trajectory comparison results of different SLAM.

In this study, in order to validate the effectiveness of the proposed method, we conducted scenario testing with mobile robots in a real scenario. As shown in Fig 10, the software of the mobile robot is implemented by the ROS (Robot Operating System) on ubuntu18.04; the vision sensor is an Intel RealSense D435I depth camera. The camera is capable of acquiring RGB and depth information with high resolution and low latency. And this camera incorporates state-of-the-art depth sensing technology. It is not only capable of performing high-precision depth measurements in both indoor and outdoor environments, but also supports applications such as dynamic object tracking and gesture recognition.

**Fig 11:**
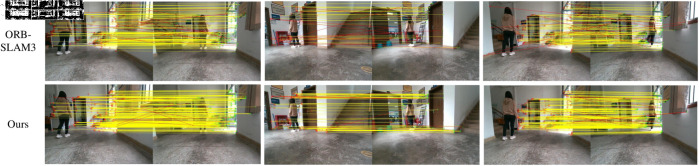
The comparison results of feature matching in real scenarios.

The feature matching performance of ORB-SLAM3 and 2HR-Net VSLAM is compared and analyzed in scenario testing within dynamic environments. In order to prevent the interference of dynamic objects, we have also integrated a YOLOv8n object detection module into the original ORB-SLAM3 framework and removed feature points within the dynamic objects. The matching results are shown in Fig 11, where the first and second rows represent the matching outcomes of ORB-SLAM3 and the algorithm proposed in this paper, respectively. In Fig 11, yellow lines represent correct feature matches, while red lines indicate incorrect matches. The comparative results reveal that the proposed algorithm has relatively fewer incorrect matches. The results show that the proposed algorithm is robust in real scenario.

## Conclusions

In this study, an improved feature detection and matching algorithm is proposed and integrated into the traditional feature-based VSLAM framework to form the 2HR-Net VSLAM system. First, the K-means clustering algorithm is applied to the L2-Net module of the R2D2 algorithm to extract feature points with high reliability and high repeatability (2HR) in a dynamic environment. The regions of dynamic objects are subsequently identified via the YOLOv8n algorithm, and the feature points located in these regions are eliminated. In this way, the quality of the feature points and the robustness of the system are enhanced. In addition, a shared Siamese network is proposed to increase the accuracy of the feature matching process. Finally, the 2HR-Net VSLAM system is constructed by integrating these improvements into a conventional VSLAM system. To verify the accuracy of the proposed method when conducting feature detection and matching in dynamic environments, some experiments are performed on the TUM dataset and in real scenarios. The experimental results show that the proposed method has a better ability to perform feature detection and matching in a dynamic environment than do the competing methods. Moreover, compared with ORB-SLAM3, the 2HR-Net VSLAM system attains improved pose estimation accuracy. The 2HR-Net VSLAM system is robust and accurate in dynamic environment.

However, during the experimental phase, we observed that the proposed method did not perform well under specific environmental conditions (such as low-texture areas). This is because in low-texture areas, the number of feature points that can be extracted is limited. After filtering, the number of feature points is further reduced, which in turn affects the feature matching results and leads to the failure of mobile robot localization. In light of this, future research will explore the integration of other feature information (such as points, lines, etc.) to optimize the localization accuracy of mobile robots. Additionally, since the research background of this study is mainly focused on indoor scenarios, further improvements in the model’s performance are needed to enhance the system’s versatility for more complex environments, such as outdoor scenes.

On the other hand, in order to reduce computational load and shorten processing time, this study only used semantic labels from object detection to determine the motion status of objects, without conducting an in-depth analysis of the actual motion status of the objects. Therefore, future research will be dedicated to more accurately assessing the motion status of objects to achieve finer motion analysis and more precise localization capabilities.

## Supporting information

S1 Text K-means clustering parameter study. This dataset presents the data from the experiment of K-means clustering parameter study. https://www.kaggle.com/datasets/wangyangcq/k-means-clustering-parameter-study.
(PDF)

S2 Text Ablation study of feature extraction and matching. This dataset presents the feature point information detected by different methods in the ablation study of 2HR feature detection and 2HR-Net feature matching. This dataset encompasses the feature point information detected by 10 distinct methods, including those proposed in this article, across four datasets from TUM. https://www.kaggle.com/datasets/wangyangcq/ablation-study-of-feature-extraction-and-matching.
(PDF)

S3 Text Evaluation of 2HR-Net VSLAM system. This dataset presents the data from the experiment of evaluation of 2HR-Net VSLAM system. This dataset records the results of trajectory detection of ORB-SLAM3 and the method proposed in this paper in the four datasets of TUM. https://www.kaggle.com/datasets/wangyangcq/evaluation-of-2hr-net-vslam-system.
(PDF)

S4 Text Real scenario evaluation. This dataset presents the data from the experiment of real scenario evaluation. This dataset documents the feature information detected by ORB-SLAM3 and the method introduced in this paper across 12 real-world scene images. https://www.kaggle.com/datasets/wangyangcq/real-scenario-evaluation.
(PDF)
