# Supplementary material for: 2HR-Net VSLAM: Robust visual SLAM based on dual high-reliability feature matching in dynamic environments
Source: PLoS One. 2025 Jul 18;20(7):e0328052. doi: 10.1371/journal.pone.0328052 (PMC12273943; doi:10.1371/journal.pone.0328052)
Supplement: S1 Text — This dataset presents the data from the experiment of K-means clustering parameter study. https://www.kaggle.com/datasets/wangyangcq/k-means-clustering-parameter-study. (PDF) [file pone.0328052.s001.pdf]

https://www.kaggle.com/datasets/wangyangcq/k-means-clustering-parameter-study
