# Supplementary material for: 2HR-Net VSLAM: Robust visual SLAM based on dual high-reliability feature matching in dynamic environments
Source: PLoS One. 2025 Jul 18;20(7):e0328052. doi: 10.1371/journal.pone.0328052 (PMC12273943; doi:10.1371/journal.pone.0328052)
Supplement: S2 Text — This dataset presents the feature point information detected by different methods in the ablation study of 2HR feature detection and 2HR-Net feature matching. This dataset encompasses the feature point information detected by 10 distinct methods, including those proposed in this article, across four datasets from TUM. https://www.kaggle.com/datasets/wangyangcq/ablation-study-of-feature-extraction-and-matching. (PDF) [file pone.0328052.s002.pdf]

https://www.kaggle.com/datasets/wangyangcq/ablation-study-of-feature-extraction-and-matching
